# Sustainable and Reusable Modified Membrane Based on Green Gold Nanoparticles for Efficient Methylene Blue Water Decontamination by a Photocatalytic Process

**DOI:** 10.3390/nano14191611

**Published:** 2024-10-08

**Authors:** Lucia Mergola, Luigi Carbone, Ermelinda Bloise, Maria Rosaria Lazzoi, Roberta Del Sole

**Affiliations:** 1 Department of Engineering for Innovation, University of Salento, Via Monteroni, 73100 Lecce, Italy; mariarosaria.lazzoi@unisalento.it; 2 National Nanotechnology Laboratory (NNL), Institute of Nanoscience CNR, c/o Campus Ecotekne, Via Monteroni, 73100 Lecce, Italy; luigi.carbone@cnr.it; 3 Institute of Atmospheric Sciences and Climate CNR (ISAC-CNR), c/o Campus Ecotekne, Via Monteroni, 73100 Lecce, Italy; ermelinda.bloise@cnr.it

**Keywords:** green synthesis, gold nanoparticles, methylene blue, photocatalysis, water remediation, sustainability, winery waste, grape marc

## Abstract

Methylene blue (MB) is a dye hazardous pollutant widely used in several industrial processes that represents a relevant source of water pollution. Thus, the research of new systems to avoid their environmental dispersion represents an important goal. In this work, an efficient and sustainable nanocomposite material based on green gold nanoparticles for MB water remediation was developed. Starting from the reducing and stabilizing properties of some compounds naturally present in Lambrusco winery waste (grape marc) extracts, green gold nanoparticles (GM-AuNPs) were synthesized and deposited on a supporting membrane to create an easy and stable system for water MB decontamination. GM-AuNPs, with a specific plasmonic band at 535 nm, and the modified membrane were first characterized by UV–vis spectroscopy, X-ray diffraction (XRD), and electron microscopy. Transmission electron microscopy analysis revealed the presence of two breeds of crystalline shapes, triangular platelets and round-shaped penta-twinned nanoparticles, respectively. The crystalline nature of GM-AuNPs was also confirmed from XRD analysis. The photocatalytic performance of the modified membrane was evaluated under natural sunlight radiation, obtaining a complete disappearance of MB (100%) in 116 min. The photocatalytic process was described from a pseudo-first-order kinetic with a rate constant (k) equal to 0.044 ± 0.010 min^−1^. The modified membrane demonstrated high stability since it was reused up to 20 cycles, without any treatment for 3 months, maintaining the same performance. The GM-AuNPs-based membrane was also tested with other water pollutants (methyl orange, 4-nitrophenol, and rhodamine B), revealing a high selectivity towards MB. Finally, the photocatalytic performance of GM-AuNPs-based membrane was also evaluated in real samples by using tap and pond water spiked with MB, obtaining a removal % of 99.6 ± 1.2% and 98.8 ± 1.9%, respectively.

## 1. Introduction

Water is fundamental for the survival of living beings, but the increasing pollution, due to the growth of industrialization and human activity, is seriously compromising the availability and healthiness of this important resource with considerable social, ecological, and economic implications. Industrial wastes and intensive agricultural treatments represent the main sources of water pollution, releasing in the environment high amounts of inorganic and organic pollutants, many of which are not biodegradable and tend to accumulate in aquatic environments, threatening human and environmental health [1,2].

Phenolic compounds, such as dyes and pesticides, are a hazardous class of organic contaminants, produced in large amounts from industrial activities. Generally, their toxicity is associated with the presence of aromatic rings in their structure that confer on these substances mutagenic and carcinogenic properties [3]. For these reasons, the research of innovative approaches to ensure drinking water purification but also efficient wastewater treatments is fundamental. Dyes, which are commonly released in the environment from the textile, pharmaceutical, and plastic industries, are not biodegradable due to their aromatic structure and their thermal and photostability [4]. Thus, their persistence in the environment can cause groundwater pollution, endangering human health. For these reasons, the capability to degrade dyes from the industrial wastewater and to put back into the industrial cycle the treated water, avoiding its environmental dispersion, would be a great achievement [5].

Methylene blue (MB) is a typical hazardous dye with carcinogenic properties widely used in industrial plants. It is well known that prolonged exposure to MB can cause serious damage to the nervous system and gastrointestinal apparatus [6]. For this reason, the research of specific methodologies to eradicate the presence of this hazardous compound from water represents an important goal [3]. Generally, biological, chemical, and physical methods are widely used for dyes removal, but these technologies showed a lot of disadvantages associated with the production of hazardous by-products that cause secondary environmental pollution. In the last few years, a considerable increase in research on new and efficient materials able to adsorb water pollutants has been observed [5,7]. Natural materials such as clays [8,9], microorganisms [10], and agricultural wastes [11,12,13], have been recently adopted as green sorbents for water remediation to remove organic and inorganic pollutants [14]. However, these approaches only move the problem but do not eliminate it. To overcome the above limit, the photodegradation technique has been successfully employed. Photodegradation is an efficient advanced oxidative process that converts harmful organic pollutants into smaller and non-toxic molecules upon light irradiation. This technique is an easy and low-cost technology with various advantages compared to the other traditional techniques, such as complete pollutant mineralization, high degradation efficiency, non-toxic byproduct, low energy consumption, and mild experimental conditions such as ambient pressure, temperature, and solar light. Metal or metal oxides are used as photocatalysts which, after adsorbing photons, accelerate the degradation processes [15,16,17,18]. Among them, recently, the application of nanotechnology to water and wastewater treatment has gained great attention. In the last few years, a lot of studies focused on noble metal nanoparticle preparation applied to water remediation have been published [19,20,21]. Gold and silver nanoparticles are the most studied systems due to their peculiar characteristics such as large surface area, chemical reactivity, biocompatibility, catalytic, and unique optical properties associated with the presence of a specific surface plasmon resonance (related to nanoparticle shape and dimension) that resulting from a typical scattering of light [22]. For these reasons, they were widely used as nanocatalysts in water remediation [3,23,24,25].

Generally, noble metal nanoparticle preparation requires the use of hazardous compounds and often expensive equipment, but recently a lot of research to exploit reducing and stabilizing agents naturally present in agricultural waste (sugars, polyphenols, tannins, anthocyanins, flavonoids, etc.) has been conducted [25,26,27,28,29]. Recently, Song and co-workers prepared green silver and gold nanoparticles using *Sargassum horneri* extract as starting materials for dye degradation in the presence of sodium borohydride (NaBH_4_), a hazardous reducing agent [30]. Green gold and silver nanoparticles were also obtained starting from the mangrove isolate *Pseudoalteromonas lipolytica*, using peptides as stabilizing agents, and they were employed for dye degradation. In particular, these photocatalytic systems showed an efficient degradation capacity of MB and congo red [31]. In another work, green gold nanoparticles capped by polyoxyethylene cholesteryl ether were synthesized, revealing high catalytic activity in the degradation of MB to leucomethylene in the presence of NaBH_4_ [32]. Alpinia nigra leaf extract was also used to synthesize green gold nanoparticles that catalyze sunlight photodegradation of pollutant dyes such as methyl orange (MO) and rhodamine B (RO), with a percentage of degradation of 83.25% and 87.64%, respectively [33]. However, in view of the increased global interest in the development of eco-sustainability processes for water purification, the use of hazardous compounds such as NaBH_4_ should be avoided. Moreover, an easy system to remove metal nanoparticles after degradation is desirable to avoid their dispersion in the environment.

Starting from this assumption, in this work, an efficient nanocomposite material based on green gold nanoparticles for MB remediation was developed. Firstly, green gold nanoparticles were synthesized using as starting materials a hydrothermal extract of Lambrusco winery grape marc (GM) waste, obtained from a local company, avoiding the use of hazardous compounds commonly used in metal nanoparticle preparation [4,25,34]. Then, green gold nanoparticles (GM-AuNPs), after characterization, were immobilized on a commercial polyvinylidene difluoride (PVDF) membrane to obtain a stable and efficient system for MB water remediation. In this way, a combination between the high photocatalytic activity of green gold nanoparticles and the high stability of PVDF membrane support occurred, favoring easy removal after its use. GM-AuNPs and the modified membrane were characterized using transmission electron microscopy (TEM), scanning transmission electron microscopy (STEM), and X-ray powder diffraction (XRD). After characterization, the modified membrane was incubated with MB solutions in the dark and under sunlight radiation without the addition of other reagents, and the degradation process was followed by UV–vis spectrophotometer. Fourier transform infrared spectroscopy (FT-IR) and total organic carbon (TOC) analysis were also used to evaluate the degradation of MB. The same experiment was performed several times to evaluate the repeatability and the stability of the nanocomposite membrane at the time. Finally, the degradation ability of the modified membrane has also been tested in the presence of other organic pollutants such as MO, RO, and 4-nitrophenol (4NP) and in real samples.

## 2. Materials and Methods

### 2.1. Materials

Gold (III) chloride trihydrate (HAuCl_4_⋅3H_2_O), MB, RO, MO, 4-NP, and filter membranes Durapore^®^ PVDF (13 mm of diameter and 0.22 µm of pore size) were acquired from Sigma-Aldrich (Steinheim, Germany). GM wastes obtained from Lambrusco winery production (2023) in the Salento area (South of Italy) were supplied from a local company (Cantina Vecchia Torre s.c.a., Leverano, Italy). Whatman 1 filters were purchased from Merck KGaA (Darmstadt, Germany). Centrifugations were conducted using a NEYA 16R High Speed refrigerated centrifuge (Giorgio Bormac s.r.l, Carpi, Italy) and a PK121 multispeed centrifuge (Thermo Electron Corporation, Waltham, MA, USA). All solutions were prepared using ultrapure water obtained using a water purification system (Human Corporation, Seoul, Republic of Korea).

### 2.2. Grape Marc Extract Preparation

GM winery wastes were sun dried for 5 days and then milled and sieved. Five grams of the obtained powder was suspended in 100 mL of ultrapure water and heated at 65 °C for 1 h [25]. After filtration with a Whatman 1 filter and centrifugation at 9000 rpm for 20 min, the extract was aliquoted and stored at −20 °C until use.

### 2.3. Synthesis of Green Gold Nanoparticles

**Scheme 1:**
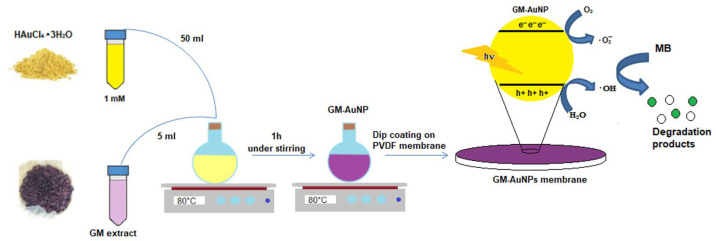
Schematic representation of GM-AuNPs-based membrane preparation.

Fifty milliliters of HAuCl_4_·3H_2_O (1 mM) aqueous solution was heated at 80 °C using a thermostatic oil bath. Then, 5 mL of GM extract was added to the solution, and the mixture was vigorously stirred for 1 h [25]. After 10 min, a color change from pale yellow to purple confirmed nanoparticle formation (Scheme 1). The synthesis was followed by using a Jasco V-660 UV–vis spectrophotometer (Jasco, Palo Alto, CA, USA) to monitor the appearance of a Plasmon resonance peak typical of AuNPs. After 1 h, the reaction was stopped and left cooling at room temperature. The purple dispersion was stored at room temperature in the dark until use.

### 2.4. Modified PVDF Membrane Preparation

Commercial PVDF membrane was activated in ethanol for 3 min, washed, and stored in ultrapure water at 4 °C. After activation, the membrane was dipped in 2 mL of green GM-AuNPs dispersion for 20 h, then washed with ultrapure water to remove residual AuNPs and stored at 4 °C in ultrapure water until further use [35]. To evaluate the amount of GM-AuNPs deposited, PVDF membrane was weighed before deposition and then, after preparation, dried at 40 °C and weighed again.

### 2.5. Characterization Studies

GM-AuNPs colloidal diluted solutions (1/10, *v*/*v*) were characterized by using a UV–vis spectrophotometer. Electrophoretic light scattering measurements, conducted with a Malvern Zetasizer nano ZS 90 (Worcestershire, UK), were evaluated on diluted samples. XRD studies of synthesized GM-AuNPs and GM-AuNPs-based membranes were performed by using a Rigaku Ultima+ model diffractometer (Rigaku, Riga, Latvia) with monochromatic Cu-kα radiation (λ = 0.154 nm), operating at 40 kV with a current of 20 mA. GM-AuNPs colloidal solution was centrifuged at 12,000 rpm (+4 °C) for 10 min and then drop-cast on a glass slide and dried in the oven at 30 °C for 24 h before analysis. All spectra were recorded in the 2θ range between 30 and 80°. The Debye–Scherrer equation applied to the peak (111) was used to evaluate the size of crystalline nanoparticles:

D = K λ/β Cos (θ)(1)

where K is the Scherrer constant, λ represents the X-ray wavelength, β is the line broadening at half the maximum intensity (FWHM), and θ is the Bragg angle. FT-IR analysis was performed using a Jasco FT-IR-660 plus spectrometer (Jasco, Palo Alto, CA, USA) applying an attenuated total reflectance (ATR) technique, with 64 scans and 2 cm^−1^ of resolution over the range of 3650–650 cm^−1^. UV–vis–NIR spectra were acquired through a Shimadzu UV-3600 i Plus. TEM analysis was performed using a JEOL JEM-1400 (JEOL, Tokyo, Japan) operating at 120 kV and equipped with a CCD camera (Gatan Orius 831). A few microliters of GM-AuNPs dispersion were drop-cast onto carbon-coated Cu grids and then dried overnight at 60 °C before investigation. The same sample deposited onto a copper grid was used for STEM measurements performed in a Zeiss Merlin SEM operating at 20 kV. SEM analysis of GM-AuNPs-loaded membranes was carried out in a field-gun emission SEM, Sigma 300 VP, Zeiss, Oberkochen, Germany, at an accelerating voltage of 15 kV, by employing a secondary electron detector under high vacuum conditions. Quantification of TOC was performed using a TOC analyzer (Shimadzu, model TOC-L series, CPH/CPN, Kyoto, Japan) on filtered samples of starting MB solution, incubated MB solution at the end of the photocatalytic process, and subtracting blanks from the process. All analyses were conducted in triplicate.

### 2.6. Photocatalytic Activity and Kinetic Studies of Modified Membrane

GM-AuNPs-based membrane photocatalytic activity was evaluated by incubating the modified membrane with 3 mL of MB solution (5.6 mg L^−1^) in a quartz cuvette 4 cm in height with 1 cm path length and kept under sunlight radiation. Solar radiation intensity was obtained from the meteorology observatory of “Orto Botanico del Salento”, Ecotekne (Lecce) (LAT 40.335 N/LONG 18.122 E). Reaction solution was monitored by a UV–vis spectrophotometer at regular intervals, recording the absorbance at 664 nm. The same experiment was conducted using a pristine PVDF membrane.

Removal % was calculated using the following equation:

Removal rate (%) = (1 − A_t_/A_0_) × 100 (2)

where A_t_ and A_0_ were associated with the absorbance at the time t and 0, respectively. The GM-AuNPs-based membrane was also incubated in the dark for 2 h, and the results obtained were compared. To study kinetic performance, linearized forms of pseudo-first-order and pseudo-second-order kinetics were used as described in the following equation:

lnC_0_/C = k_1_t (3)

t/C_t_ = 1/k_2_C_0_^2^ + 1/C_0_t (4)

where C_0_ and C_t_ represent the initial concentration and the concentration at the time t of dye pollutant, respectively, and k_1_ and k_2_ the pseudo-first-order and pseudo-second-order rate constants, respectively [36,37,38]. The same experiment was also conducted on real samples. In particular, tap and pond water (obtained from a local marshy area) were filtered, spiked with MB (5.6 mg L^−1^) and subjected to sunlight radiation in the presence of the modified membrane. To evaluate the specificity of the system, the modified membrane was also incubated separately under sunlight radiation with 5.6 mg L^−1^ of MO, RO, and 4NP solutions. All experiments were conducted in triplicate. All experiments were conducted in triplicate.

### 2.7. Reusability of Modified Membrane in MB Degradation

The modified membrane was incubated in 3 mL of MB solution (5.6 mg L^−1^) in a quartz cuvette 4 cm in height with 1 cm path length and kept under sunlight radiation for 2 h. The absorbance of the peak at 664 nm was monitored at regular intervals by a UV–vis spectrophotometer. Then, the modified membrane was reused for other degradation processes, without any treatment. The same experiment was repeated 20 times in a period of three months.

## 3. Results and Discussion

### 3.1. Synthesis and Characterization of Green Gold Nanoparticles

Grape marc (GM) represents a high-value waste obtained from the winery industry rich of relevant evaluable products such as tannins, polyphenols, pigments, and unfermented sugars with important reducing and stabilizing properties [34]. The high solubility of these substances in hot water promotes their hydrothermal extraction, and they can be used as starting materials for green noble metal nanoparticle preparation instead of toxic substances generally employed [25]. In detail, our attention was focused on the use of GM extract, obtained at 65 °C, to synthesize green gold nanoparticles with a specific plasmonic band, associated to nanoparticle dimensions, that confer notable photocatalytic properties when opportunely irradiated.

**Figure 1:**
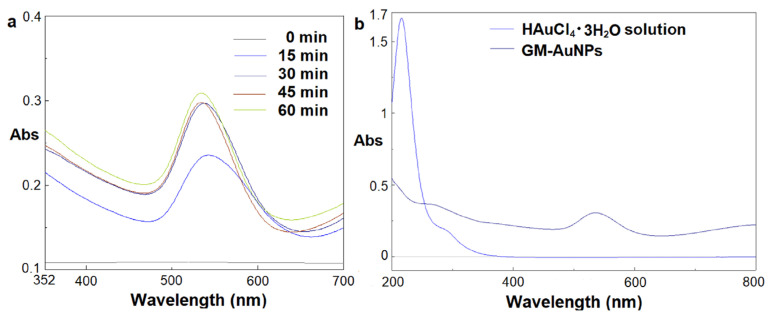
UV–vis spectra of crude reaction (diluted 1/10; *v*/*v*) at regular intervals of time (**a**) and comparison between UV–vis spectra of HAuCl_4_·3H_2_O (1 mM; diluted 1/10; *v*/*v*) solution and synthesized GM-AuNPs (diluted 1/10; *v*/*v*) (**b**).

**Figure 2:**
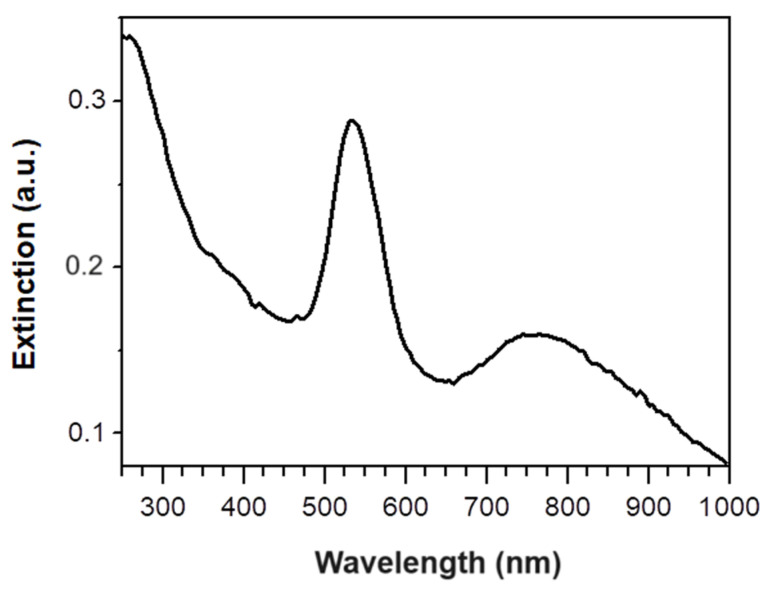
UV–vis near-infrared reflectance (NIR) extinction spectrum of GM-AuNPs.

An easy and fast methodology was employed to synthesize GM-AuNPs, adding the extract to an auric solution heated at 80 °C. After 10 min of vigorous stirring, the color of the solution changes from pale yellow to purple, indicating the reduction of Au^3+^ to Au^0^. Nanoparticle formation was monitored by a UV–vis spectrophotometer, analyzing diluted samples of crude reaction every 15 min. As it can be seen in Figure 1a, a plasmonic band at 545 nm appears after 15 min, shifting to 535 nm after 1 h. The results obtained confirmed gold nanoparticle formation from GM extract through different steps. Initially, (activation step) Au^3+^ ions were reduced to Au^0^, and the nucleation occurred. This step was followed by the formation of larger nanoparticles (growth step), confirmed from the plasmonic band at 545 nm obtained after 15 min. Then, in the final step, all nanoparticles take on the shape energetically favored, reducing their dimension, as confirmed by the plasmonic band shifted at 535 nm (Figure 1a) [39]. Overlapping the UV–vis spectra of diluted auric solution (1 mM), used for reaction, and crude reaction obtained after 1 h, the disappearance of the typical peak of gold ions (216 nm) and the appearance of a plasmonic band (535 nm) were observed, demonstrating nanoparticle formation (Figure 1b). Moreover, a new signal was observed over 600 nm; indeed, a band at 760 nm, probably due to in-plane dipole resonance of triangular NPs, appears in the extinction spectrum (Figure 2).

**Figure 3:**
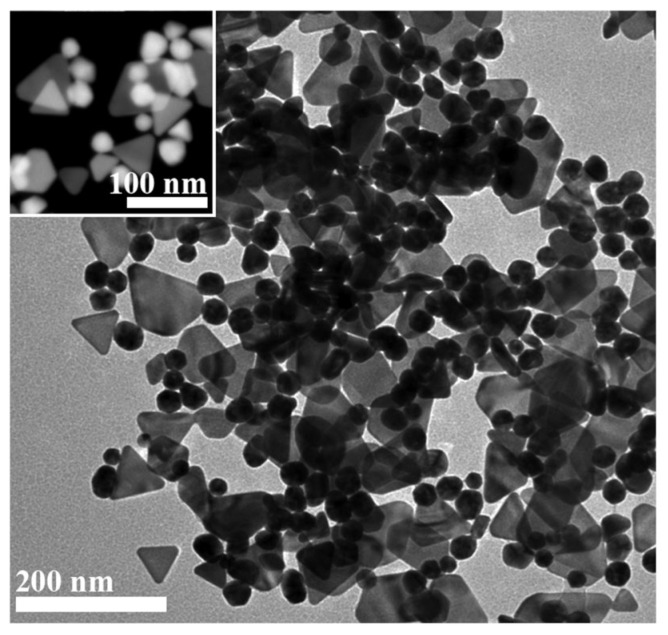
TEM and STEM (inset) analysis of GM-AuNPs.

These data were confirmed from TEM analysis that revealed the evident presence of two species of crystalline colloidal NPs, respectively, quasi-two-dimensional triangular platelets as large as 75–80 nm showing smoothed corners that reasonably evolve from (truncated) hexagonal profiles, as confirmed by many hexagonal shapes, and 30 nm in diameter irregularly spherical twinned NPs (Figure 3). STEM analysis confirmed TEM observations (see inset in Figure 3). The GM extract contains a wide variety of bioactive compounds, particularly polyols with the assistance of other reducing and passivating agents, that are reasonably supposed to act as reducing agents throughout the synthesis and function as NP stabilizers (Figure 3) [40].

**Figure 4:**
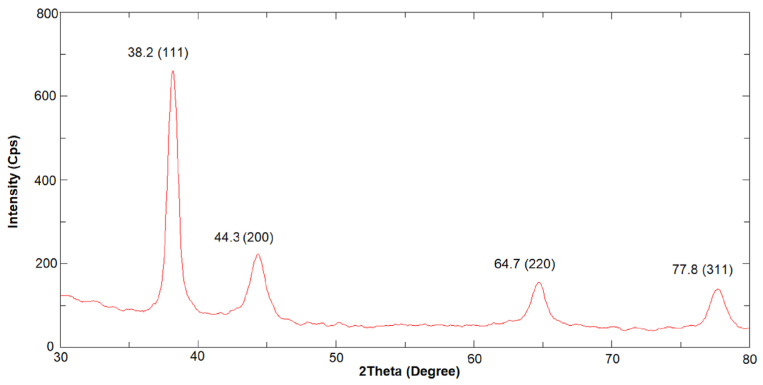
XRD pattern of GM-AuNPs deposited on a glass slide.

To further confirm the crystalline nature of synthesized GM-AuNPs, XRD analysis was also conducted in the range of 30–80° 2θ angle. As it can be seen in Figure 4, four Bragg reflection peaks at 38.2°, 44.3°, 64.7°, and 77.8° were obtained that correspond to Miller indexes of (111), (200), (220), and (311), respectively.

The results obtained reveal a good match with AuNPs standard diffraction pattern of face-centered cubic gold, confirming the presence of pure crystalline gold nanoparticles. The diffractogram shows the (111)-facet preferential growth of Au platelets. It can be concluded that gold nanoparticles with a crystalline structure, obtained from grape marc extract, were successfully synthesized. Moreover, zeta-potential analysis was also conducted, revealing a negative charge of GM-AuNPs with a zeta-potential equal to −32.6 ± 5.5 mV (Figure S1).

### 3.2. Dip Coating of PVDF Membrane in GM-AuNPs Dispersion

**Figure 5:**
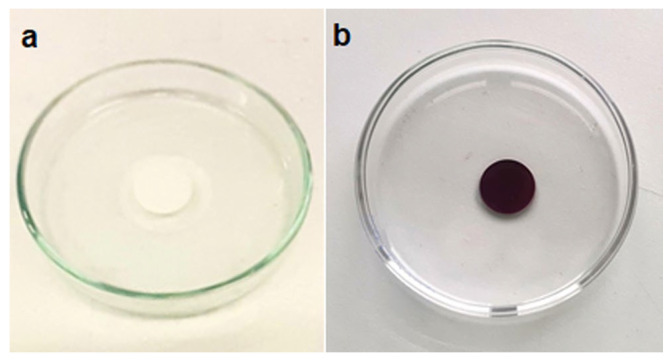
Comparison between the membrane before (**a**) and after GM-AuNPs deposition (**b**).

To develop a simple and fast system to test the photocatalytic activity of GM-AuNPs towards MB, gold nanoparticles were deposited by dip coating on a PVDF commercial membrane (Scheme 1). Generally, the use of polymeric supports is desirable to avoid gold nanoparticle aggregation due to van der Waals interactions established between adjacent nanoparticles and to facilitate easy removal after water purification, avoiding centrifugation procedures, and favoring its applicability in real systems. However, the high hydrophobicity of pristine PVDF membranes due to the fluorocarbon groups (-CF_2_) restricts their use. For this reason, an easy and fast hydrophilic activation in ethanol was required before nanoparticle deposition. The infiltration of ethanol determines a temporary change from hydrophobic to hydrophilic. In this way, electrostatic interactions between the activated PVDF membrane and GM-AuNPs negatively charged can occur [35]. As it can be seen in Figure 5, a uniform layer of nanoparticles was obtained. During deposition, it is very important to leave both faces of the membrane free of contact from vial surfaces to obtain the same deposition layer on both membrane sides. The amount of GM-AuNPs deposited was evaluated by weighting the membrane before and after dip coating, obtaining an amount of deposited GM-AuNPs equal to 158 ± 10 μg cm^−2^ of membrane.

**Figure 6:**
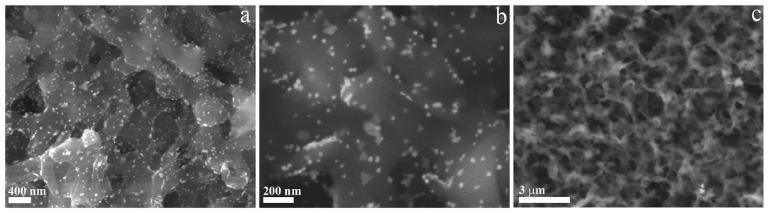
SEM analysis of GM-AuNPs-based membrane at lower (**a**) and larger magnification (**b**) and of pristine membrane (**c**).

Figure 6 reports SEM micrographs of GM-AuNPs-based membrane at lower and larger magnification (see Figure 6a,b) and of pristine membrane (Figure 6c). GM-AuNPs are evenly dispersed (in length and depth) within the membrane on the scale of many tens of micrometers, without the formation of aggregates. The nanoparticles surface can thus be uniformly exposed to adsorbate interactions. Moreover, XRD analysis conducted on the GM-AuNPs-based membrane confirmed the presence of GM-AuNPs previously characterized. Indeed, reflection peaks at 38.3°, 44.4°, 64.9°, and 77.8°, corresponding respectively to indexes of (111), (200), (220), and (311), similarly to the XRD pattern of GM-AuNPs, were observed (Figure S2). Moreover, the width of the most intense peak (111) was used to evaluate the size of crystalline nanoparticles applying the Debye–Scherrer equation. The results obtained showed an average size of 19.3 ± 5.5 nm, very near to the results obtained from TEM analysis for smaller nanoparticles.

### 3.3. Photocatalytic Studies

**Figure 7:**
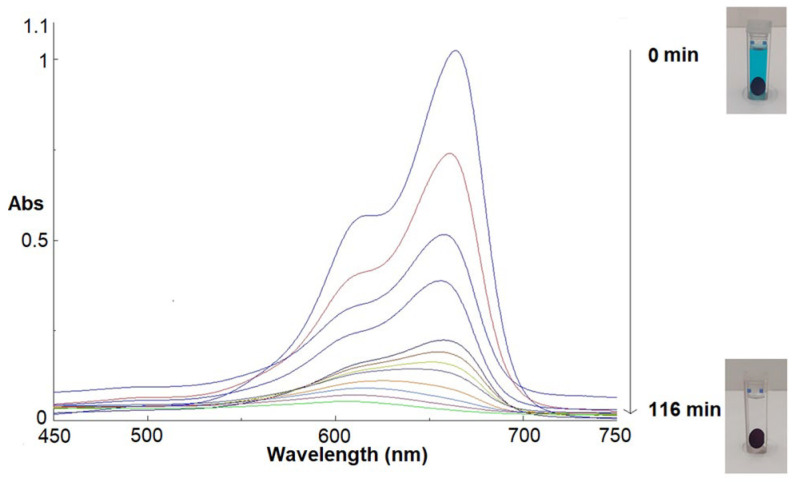
UV–vis absorption spectra of MB photodegradation using GM-AuNPs-based membrane under sunlight radiation at regular intervals of time.

To evaluate the photocatalytic effect of GM-AuNPs deposited on the activated membrane, different experiments were conducted under sunlight exposure (904 ± 33 W m^−2^), chosen to mimic real conditions. In detail, a pristine and a modified membrane were incubated with an aqueous solution of MB (5.6 mg L^−1^) and exposed to sunlight to evaluate their performance. The UV–vis spectrum of MB presents two characteristic peaks at 611 and 664 nm associated with the conjugated structure of the N-S heterocycle group. When a GM-AuNPs-based membrane was used, a gradual reduction in MB adsorption peak intensity was observed, with its complete disappearance after 116 min (Figure 7). On the contrary, MB solution, exposed to sunlight without adding the membrane, showed a good stability with a removal rate lower of 4%.

**Figure 8:**
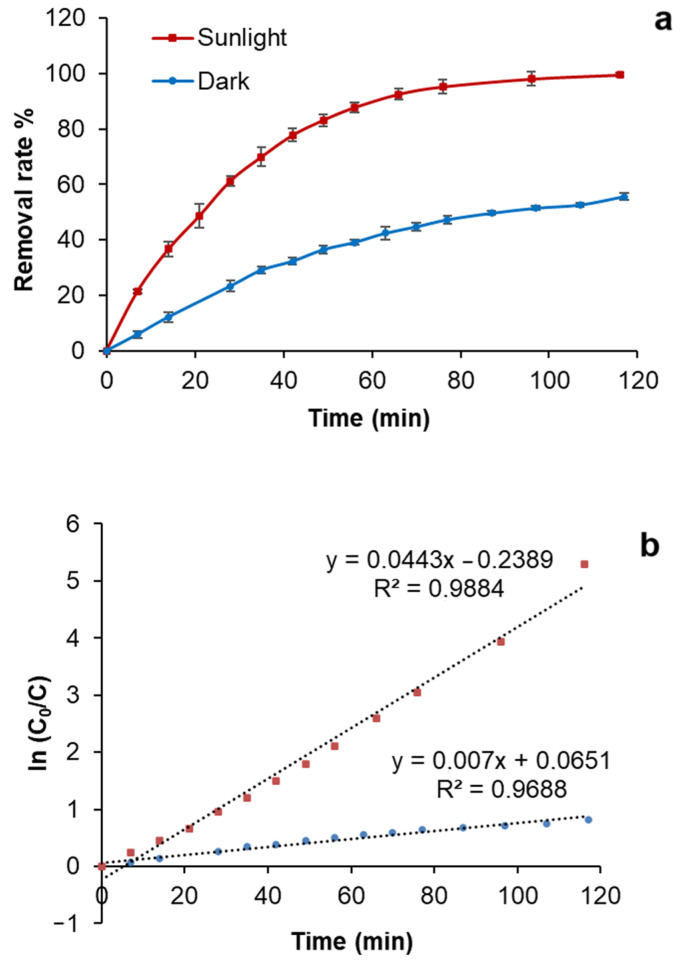
Kinetic performance (**a**) and pseudo-first-order kinetic linearized model (**b**) of GM-AuNPs-based membrane in the dark and under sunlight radiation.

To understand the influence of sunlight radiation, the same experiment was also conducted in the dark. As it can be seen in Figure 8a, comparing the performance of the modified membrane in the dark and under sunlight radiation, a significant difference can be observed with a relevant lower removal percentage observed in the dark. Probably, only an adsorption process of the membrane occurs in the dark, while the sunlight also promotes a photodegradation process mediated by GM-AuNPs that allows the complete removal of the MB. Generally, photodegradation of organic pollutants follows pseudo-first-order kinetics [36,37,38,39,40,41]. As it can be seen in Figure 8b, plotting the ln(C_0_/C) versus t, a linear regression was obtained with k_1_ of 0.044 ± 0.011 min^−1^ when the experiment was conducted under sunlight radiation, which was 5.6 times higher than the k_1_ value obtained in the dark (0.007 ± 0.002 min^−1^). These results confirmed the influence of sunlight radiation on the MB degradation process, mediated by GM-AuNPs deposited on the membrane. Analyzing these data, it can be concluded that MB degradation occurs through a combined adsorption and photodegradation effect, also found in other works [39].

Indeed, an adsorption of MB on the modified membrane could be a possible initial process, allowing a close contact between MB and making the photocatalytic degradation easier. Concerning AuNPs, depending on the wavelength excitation region, two optical contributions can be activated towards photocatalysis, namely intraband and interband excitations. In the former case, the stimulation of a localized surface plasmon resonance occurs, involving electronic transitions within the sp-band. In the latter case, transitions from d- to sp-bands are associated. In the case of gold, interband transitions relate to short wavelength regions, usually below 450 nm [42]. The two optical contributions promote two different catalytic paths, exciting different charge carriers; in the case of excitation of localized surface plasmon resonance, high-energy (hot) electrons can be created (5) having an energy higher than the Fermi level; this could be transferred to an adsorbate or to oxygen/water molecules to produce reactive radicals (6). In the case of interband excitation, instead, very energy-deep holes showing low potential (below Fermi energy) can be generated (9); these are more available for very oxidative paths. For instance, the formation of highly reactive superoxide (6) and hydroxyl free radicals (7) is most likely, and they are responsible for MB photocatalytic degradation (9) and (10).

GM-AuNPs + h+ → GM-AuNPs (e−/h+)(5)

e− + O_2_ → ·O_2_^−^ (6)

h+ + MB → MB+(7)

h+ + OH− → OH(8)

·OH + MB dye → CO_2_ + H_2_O (9)

·O_2_^−^ + MB dye → CO_2_ + H_2_O (10)

In general, it is not straightforward to claim which mechanism is the more likely of the two. Indeed, further studies should be carried out to validate this hypothesis. However, in addition to CO_2_ and H_2_O, minor degradation products could also be formed [3].

**Figure 9:**
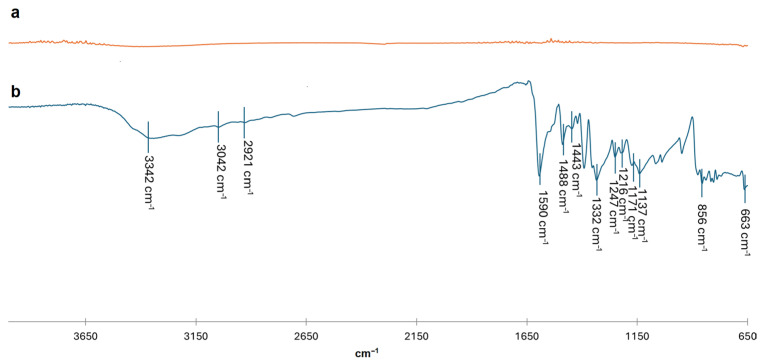
FT-IR spectra of MB solution before (**b**) and after (**a**) photocatalytic degradation.

Furthermore, a (photo)thermal input, a critical factor in catalysis, cannot be completely ruled out; indeed, because of the characteristic shape of triangles, thermal hotspots are created around metal apexes where an enhancement of the electromagnetic field occurs and of the electron transfer strength to adsorbed molecules [43]. The presence in the reaction medium of these reactive species probably determined the degradation of MB initially absorbed into the membrane, causing firstly the breakage of the N-CH_3_ bond and then the aromatic ring. All fragments produced are further attacked by this reactive species and finally degraded into smaller, untraceable byproducts [3]. To support these hypotheses, FT-IR and TOC analyses of MB solutions before and after sunlight radiation were also conducted. As it can be seen in Figure 9b, all characteristic peaks of MB functional groups can be observed. It can underline the presence of an adsorption peak at 1590 cm^−1^ associated with C=C framework vibration and C=N stretching vibration of the benzene ring. Moreover, the adsorption peaks at 1488 and 1443 cm^−1^ were also correlated to the C=C vibration. At 1247 cm^−1^ it can be seen a characteristic peak related to C=S stretching vibration. Aromatic C-H in plane bending vibration can be noted at 1216, 1171, and 1137 cm^−1^, while out-plane bending vibration peaks were also found at 856 and 663 cm^−1^. The broad peak at 3342 cm^−1^ was associated with the O-H stretching vibration of water [44]. After photocatalytic degradation, the FT-IR spectrum of degradation products (Figure 9a) does not show significant peaks associated with the presence of organic byproducts; thus, this technique is not sufficient to monitor them. Moreover, it was interesting to note bubble formation during the photocatalytic process, suggesting the formation of gaseous species.

To complete the characterization of degradation products, TOC analysis was conducted. Results obtained confirmed a degradation % of MB to CO_2_ and H_2_O equal to 75 ± 5%. It can be concluded that MB was mainly degraded into CO_2_ and H_2_O in 116 min with the presence of minor amounts of organic byproducts not detectable with FT-IR and UV–vis analysis.

**Figure 10:**
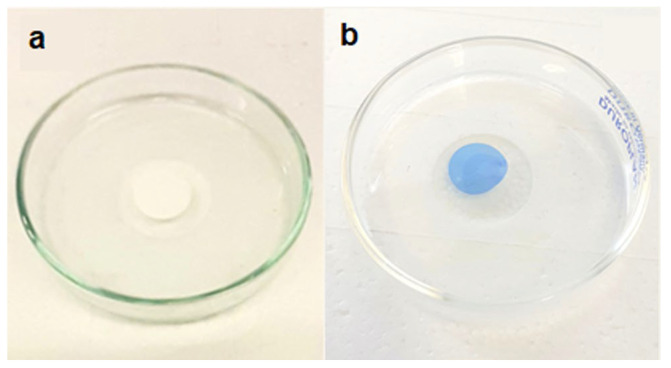
Pristine PVDF membrane before (**a**) and after sunlight incubation with MB solution (**b**).

To corroborate the synergistic effect of adsorption and photodegradation processes in MB degradation, a pristine PVDF membrane was incubated with MB solution (5.6 mg L^−1^) and exposed to sunlight. After 2 h of incubation, 90% of MB was removed from the solution but through an adsorption process, as can be seen from the blue coloration of the membrane after incubation (Figure 10).

**Figure 11:**
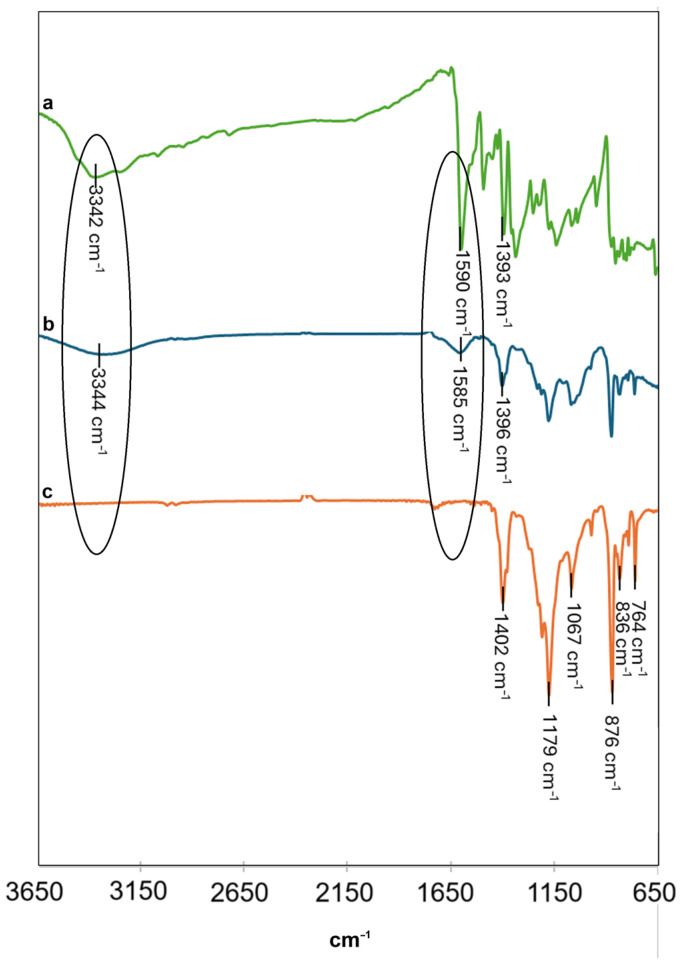
FT-IR spectra of MB solution (**a**), pristine membrane (**b**), and GM-AuNPs-based membrane (**c**) after sunlight incubation for 2 h with MB solution.

To confirm the above hypothesis, in Figure 11, FT-IR spectra of pristine and modified membranes after sunlight radiation were reported and overlapped with the MB solution spectrum.

As it can be seen, the pristine PVDF membrane after sunlight incubation showed the presence of peaks at 3344 and 1585 cm^−1^ associated with the O-H stretching vibration of water and the C=C framework vibration and the C=N stretching vibration of the benzene ring, respectively (Figure 11b), that confirmed the presence of MB adsorbed on the membrane. On the contrary, no peaks associated with MB were found in the FT-IR spectrum of the GM-AuNPs-based membrane after sunlight incubation (Figure 11c), confirming the occurred MB degradation. Optimized conditions were also used to evaluate the performance of the modified membrane in the presence of higher MB concentrations. In Table 1, a comparison at different MB concentrations of degradation % measured after 2 h of sunlight radiation was reported: increasing MB concentration, the efficiency of the GM-AuNPs-based membrane decreases. The reduction in efficiency of the modified membrane in the presence of a higher MB concentration was probability due to the higher MB adsorption at a lower concentration that favors direct contact with GM-AuNPs and consequently its photocatalytic degradation.

To evaluate the applicability in real samples, the performance of the GM-AuNPs-based membrane in MB degradation was also evaluated in tap water and pond water, obtained from a local marshy area. The samples, after filtration, were spiked with MB (5.6 mg L^−1^) and subjected to photocatalytic degradation under sunlight irradiation (692 ± 20 W m^−2^) for 116 min, obtaining a removal % of 99.6 ± 1.2% and 98.8 ± 1.9%, respectively.

### 3.4. Modified Membrane Behavior with Other Organic Pollutants

**Figure 12:**
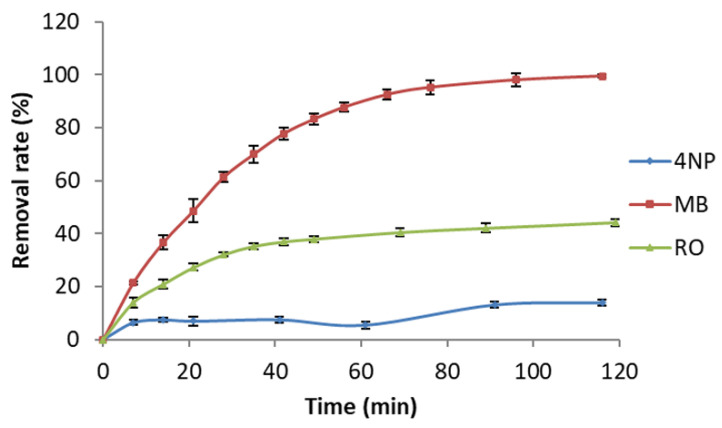
Comparison of kinetic performance of GM-AuNPs-based membrane towards other pollutants.

Photocatalytic performances of the GM-AuNPs-based membrane were also tested using other dyes (methyl orange and rhodamine) and 4-nitrophenol, a typical pesticide widely used as a model. As it can be noted in Figure 12, a total degradation occurred only with MB, underlining the selectivity of the system. The experiment conducted, using RO as a pollutant, showed a decrease in the principal peak signal (554 nm) lower than 45%. However, after 2 h of sunlight radiation, the membrane was stored in 3 mL of ultrapure water, and an evident release of RO in solution (78% of adsorbed RO), monitored by spectrophotometric analysis, was observed. Thus, it can be assumed that using RO, an adsorption process, also favored by its positive charge, occurred. Following the principal absorption peaks of MO and 4NP solution (465 and 317 nm, respectively), no significant reduction after sunlight radiation was observed. Probably, the negative charge of MO produces electrostatic repulsion between MO and GM-AuNPs, negatively charged, hindering the direct contact with the nanoparticles, essential to trigger the photocatalytic reaction, as demonstrated also in other works [39].

According to the typical adsorption peak at 317 nm, 4NP in water is present in neutral form, reducing its contact with gold nanoparticles. Kinetic data were processed using pseudo-first-order and pseudo-second-order kinetic models in linear form, and the results were reported in Table 2. It is interesting to note that only using MB a linear fitting with a pseudo-first-order kinetic, generally observed in photodegradation processes, can be obtained (R^2^ = 0.9884) [36,41]. Instead, the incubation of the GM-AuNPs-based membrane with RO, 4NP, and MO showed a prevalent linearity with a pseudo-second-order kinetic in accordance with the correlation coefficient obtained (Table 2).

### 3.5. Reusability and Stability

**Figure 13:**
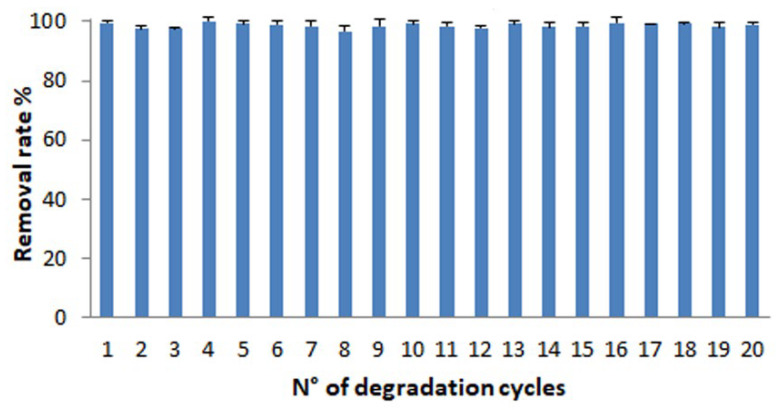
Removal rate (%) obtained after reuse of modified membrane for 20 cycles.

The potential reusability of the modified membrane represents the principal requirements to investigate the real applicability of the developed system in water remediation. To evaluate the performance over time, the same modified membrane was incubated with MB solution (5.6 mg L^−1^) 20 times over a period of 3 months, and no loss of efficiency in MB degradation was observed, as it can be seen in Figure 13. It is interesting to note that no washing step between each incubation was performed, confirming that MB photodegradation has occurred. Indeed, if the disappearance of MB in solution was due only to an adsorption process, after a few cycles, a saturation of the membrane would have happened.

In a recent work, Singh and co-workers developed green gold nanoparticles used for MB degradation. Reusability performance of similar systems showed a reduction in degradation performance from 94% to 74% in 5 cycles [39]. Moreover, in another work that uses green gold nanoparticles embedded into magnetic carbon nanocages, it requires different washing steps before each reuse, which was tested only 8 times [45]. Thus, a good nanocomposite material for MB water remediation was developed. Indeed, a high efficiency of the modified membrane was also observed in the last cycle of incubation (20th cycle), which corroborates the photodegradation hypothesis previously advanced. Moreover, the high reusability determined a significant cost reduction, although gold nanoparticles were used.

## 4. Conclusions

In this work, an easy and low-cost sustainable system for MB water remediation was developed. Indeed, although an expensive gold salt was used for GM-AuNPs preparation, the absence of other reagents in their green preparation and the high reusability of the same modified membrane without losing degradation capability lead to an evident cost reduction. The GM-AuNPs-based membrane developed showed a good power in MB remediation also in real samples and an efficiency of reuse tested for 20 cycles of incubation. TOC analysis demonstrated a degradation % of MB to CO_2_ and H_2_O equal to 75 ± 5%. Probably, reactive species formed during GM-AuNPs photoactivation determined also the production of other minor degradation products undetectable with FT-IR and UV–vis analysis. Moreover, no sophisticated instrumentation was required, making their production and use highly sustainable. Starting from the important results obtained in this work, it would be interesting to study in the future all specific photoinduced mechanisms involved in MB photodegradation and evaluate the real possibility to exploit the peculiarity of the developed membrane in industrial wastewater treatment. Moreover, considering the excellent physical characteristics of GM-AuNPs obtained using a green route, the possible application in medicine could also be explored. Indeed, the presence of quasi-two-dimensional triangular platelets as large as 75–80 nm, in addition to irregularly spherical twinned nanoparticles, confers to the important NIR-absorbing colloidal metal nanoparticle solution photothermal characteristics that would make them ideal in phototherapy treatments.

## Figures and Tables

**Table 1:** Degradation % obtained incubating GM-AuNPs-based membrane with different MB concentrations.

MB Concentration (mg L^−1^)	Degradation % ± SD (%)
5.6	99.5 ± 0.7
15	86.8 ± 1.5
30	73.8 ± 2.1
40	63.7 ± 2.3

**Table 2:** Comparison of pseudo-first-order and pseudo-second-order parameters obtained fitting the experimental data after incubation of GM-AuNPs-based membrane with MB and other pollutants.

Polluttants	Pseudo-First-Order		Pseudo-Second-Order	
	k_1_ (min^−1^)	R^2^	K_2_ (L mg^−1^ min^−1^)	R^2^
MB	0.0443	0.9884	0.8340	0.5179
RO	0.0043	0.7359	0.0822	0.9974
4NP	0.0010	0.8184	0.1640	0.9991
MO	0.0003	0.3387	11.891	0.9994

## Data Availability

Data are contained within the article.

## Supplementary Materials

The following supporting information can be downloaded at: https://www.mdpi.com/article/10.3390/nano14191611/s1, Figure S1. Z-potential of GM-AuNPs; Figure S2. XRD pattern of GM-AuNPs-based membrane.
