# Supplementary material for: Sustainable and Reusable Modified Membrane Based on Green Gold Nanoparticles for Efficient Methylene Blue Water Decontamination by a Photocatalytic Process
Source: Nanomaterials (Basel). 2024 Oct 8;14(19):1611. doi: 10.3390/nano14191611 (PMC11478303; doi:10.3390/nano14191611)
Supplement: Supplementary file 1 [file nanomaterials-14-01611-s001.zip › nanomaterials-3206686-supplementary.pdf]

Sustainable and Reusable Modified Membrane Based on Green Gold Nanoparticles for Efficient Methylene Blue Water Decontamination by a Photocatalytic Process

Lucia Mergola [1,*], Luigi Carbone [2], Ermelinda Bloise [3], Maria Rosaria Lazzoi [1] and Roberta Del Sole [1,*]

[1] Department of Engineering for Innovation, University of Salento, Via Monteroni, 73100 Lecce, Italy;
mariarosaria.lazzoi@unisalento.it
[2] National Nanotechnology Laboratory (NNL), Institute of Nanoscience CNR c/o Campus Ecotekne,
Via Monteroni, 73100 Lecce, Italy; luigi.carbone@cnr.it
[3] Institute of Atmospheric Sciences and Climate, ISAC-CNR, c/o Campus Ecotekne, Via Monteroni,
73100 Lecce, Italy; ermelinda.bloise@cnr.it
* Correspondence: lucia.mergola@unisalento.it (L.M.); roberta.delsole@unisalento.it (R.D.S.)

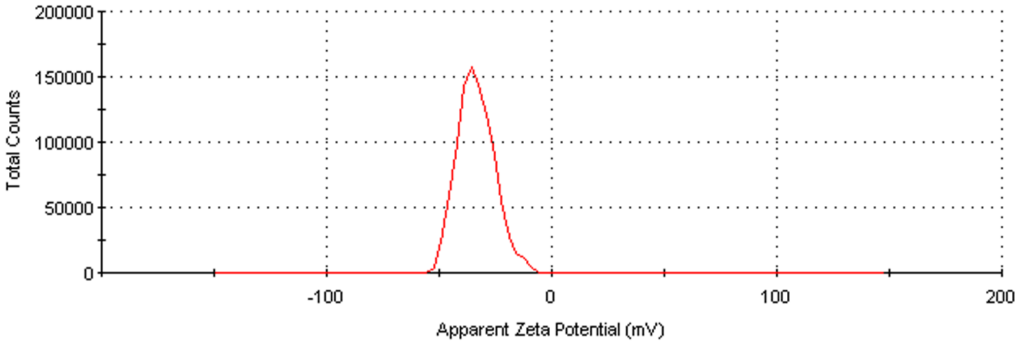

Figure S1. Z-potential of GM-AuNPs

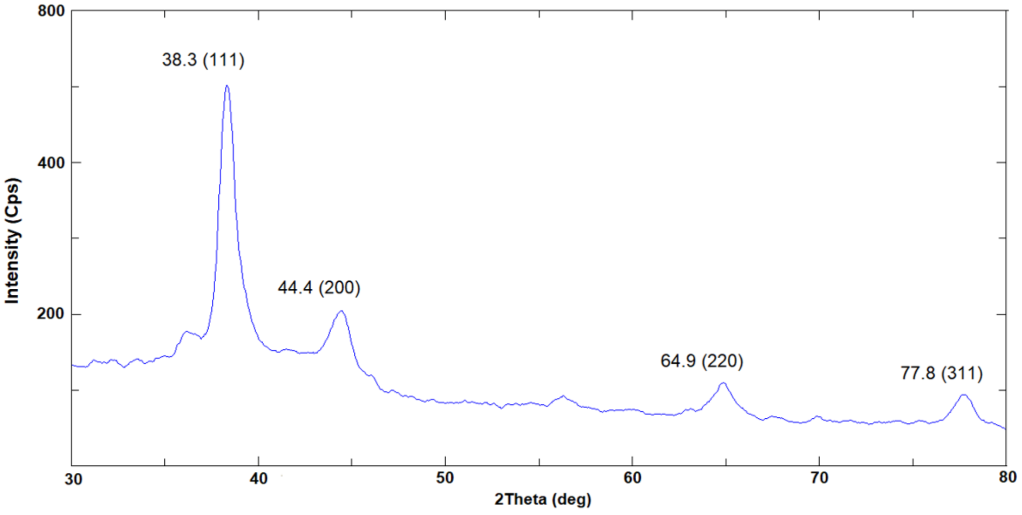

Figure S2. XRD pattern of GM-AuNPs-based membrane
